# Drought Stress Acclimation Imparts Tolerance to Sclerotinia sclerotiorum and Pseudomonas syringae in Nicotiana benthamiana

**DOI:** 10.3390/ijms14059497

**Published:** 2013-05-02

**Authors:** Venkategowda Ramegowda, Muthappa Senthil-Kumar, Yasuhiro Ishiga, Amita Kaundal, Makarla Udayakumar, Kirankumar S. Mysore

**Affiliations:** 1 Plant Biology Division, Samuel Roberts Noble Foundation, Ardmore, OK 73401, USA; E-Mails: skmuthappa@noble.org (M.S.-K.); yishiga@noble.org (Y.I.); akaundal@noble.org (A.K.); 2 Department of Crop Physiology, University of Agricultural Science, GKVK, Bangalore 560065, India; E-Mail: udayakumar_m@yahoo.com

**Keywords:** *Nicotiana benthamiana*, drought acclimation, disease tolerance, reactive oxygen species

## Abstract

Acclimation of plants with an abiotic stress can impart tolerance to some biotic stresses. Such a priming response has not been widely studied. In particular, little is known about enhanced defense capacity of drought stress acclimated plants to fungal and bacterial pathogens. Here we show that prior drought acclimation in *Nicotiana benthamiana* plants imparts tolerance to necrotrophic fungus, *Sclerotinia sclerotiorum*, and also to hemi-biotrophic bacterial pathogen, *Pseudomonas syringae* pv. *tabaci. S. sclerotiorum* inoculation on *N. benthamiana* plants acclimated with drought stress lead to less disease-induced cell death compared to non-acclimated plants. Furthermore, inoculation of *P. syringae* pv. *tabaci* on *N. benthamiana* plants acclimated to moderate drought stress showed reduced disease symptoms. The levels of reactive oxygen species (ROS) in drought acclimated plants were highly correlated with disease resistance. Further, *in planta* growth of *GFPuv* expressing *P. syringae* pv. *tabaci* on plants pre-treated with methyl viologen showed complete inhibition of bacterial growth. Taken together, these experimental results suggested a role for ROS generated during drought acclimation in imparting tolerance against *S. sclerotiorum* and *P. syringae* pv. *tabaci*. We speculate that the generation of ROS during drought acclimation primed a defense response in plants that subsequently caused the tolerance against the pathogens tested.

## 1. Introduction

Apart from thriving under several abiotic stress factors, plants must also defend themselves from pathogen attack [1,2]. When exposed to multiple stress conditions plants activate specific and unique stress responses [3,4]. Plants response to complex biotic and abiotic stress conditions include expression of stress-inducible genes that function in stress tolerance, activation of signaling cascades, production of reactive oxygen species (ROS) and accumulation of hormones such as salicylic acid (SA), ethylene, jasmonic acid (JA) and abscisic acid (ABA) [5–7].

Plant adaptation to any stress situation has been suggested to be mediated by both basal and induced defenses [8,9]. The effectiveness of this basal resistance can be enhanced by pre-exposing plants to specific mild abiotic or biotic stimuli before imposing the full strength of respective stress. For example, prior exposure of plants to cold and drought stress has resulted in more stronger and successful response to the subsequent cold or drought stress, a phenomenon known as acclimation [10,11]. Initial cold acclimation of plants has increased the tolerance to subsequent cold stress in different species [12–15]. Prior exposure of *Arabidopsis* plants to drought stress or ABA resulted in freezing tolerance in those plants [16]. In wheat, pre-anthesis high-temperature acclimation alleviated damage to the flag leaf caused by post-anthesis heat stress [17]. Initial acclimation of sunflower seedlings to mild temperature provided better tolerance to subsequent high temperature and these heat acclimated seedlings also showed oxidative stress tolerance [18]. Chemical priming of *Arabidopsis* plants with the non-protein amino acid β-aminobutyric acid (BABA) increased drought and salt stress tolerance through accelerated stress gene expression and stomatal closure mediated by ABA, suggesting the possibility of increasing plant tolerance to abiotic stresses through effective priming of the pre-existing defense pathways [19].

Similarly, prior exposure of plants to biotic stress stimuli resulted in stronger and faster defense response upon subsequent exposure to pathogens, a phenomenon known as priming [20]. Prior pathogen infection or treatments with natural or synthetic compounds like SA, riboflavin, thiamine, menadione, 2,6-dichloroisonicotinic acid (INA), benzothiadiazole (BTH), BABA, or phosphite (Phi) has been shown to prime defense against subsequent pathogen challenge by mostly potentiating SA defense signaling [21–29]. Numerous studies have attempted to understand the components of primed defense. Treatment of *Arabidopsis* leaves with Phi resulted in enhanced accumulation of H_2_O_2_ not only at the site of *Phytophthora cinnamomi* inoculation but also in cells away from the inoculation [28], indicating the role of ROS in priming the defense response.

In nature, plants are simultaneously exposed to various biotic and abiotic stresses. Plants have mechanisms to survive in such complex environmental conditions [30]. In recent years, there are emerging reports to show that enhanced defense response of abiotic stress acclimated/treated plants to pathogens and *vice versa* [31]. For example, exposure of *Arabidopsis* plants to ozone or UV irradiation has induced resistance to virulent phytopathogenic *Pseudomonas syringae* strains [32] and to tobacco mosaic virus in tobacco [33]. Similarly, infection of various host plants with cucumber mosaic virus improved drought tolerance and also enhanced freezing tolerance in *Beta vulgaris* [31]. However, very little is known about how prior exposure of plants to abiotic stress improves the capacity of plants to effectively respond to biotic stress.

Upon successful recognition of avirulent pathogens, plants elicit a biphasic ROS accumulation with a rapid but weak transient first phase followed by a massive and prolonged second phase. The second phase increase in ROS, also called oxidative burst, is highly correlated with bacterial disease resistance [34]. Regulation of several abiotic stress signaling pathways is also associated with ROS [35]. Therefore, the generation of ROS has been proposed as a key process that is shared between biotic and abiotic stress responses [35,36]. Rapid production and tight regulation of the steady-state levels of ROS plays a pivotal role in both abiotic stress signaling and disease resistance responses [37,38]. Rapid generation of superoxide (O_2_^−^) and accumulation of hydrogen peroxide (H_2_O_2_) is a characteristic early feature of cell death or hypersensitive response (HR) following perception of pathogen signals [39–41]. In this study, we used *Nicotiana benthamiana* to investigate the effect of drought acclimation on subsequent challenge with necrotrophic fungus, *Sclerotinia sclerotiorum* and hemi-biotrophic bacterial pathogen, *P. syringae* pv. *tabaci*. Upon infection, drought acclimated plants showed less disease-induced cell death when compared to control non-acclimated plants. This correlated with the levels of ROS in the drought acclimated plants.

## 2. Results and Discussion

### 2.1. Drought Acclimated *N. benthamiana* Plants Showed Increased ABA and Root-Shoot Ratio

**Figure 1:**
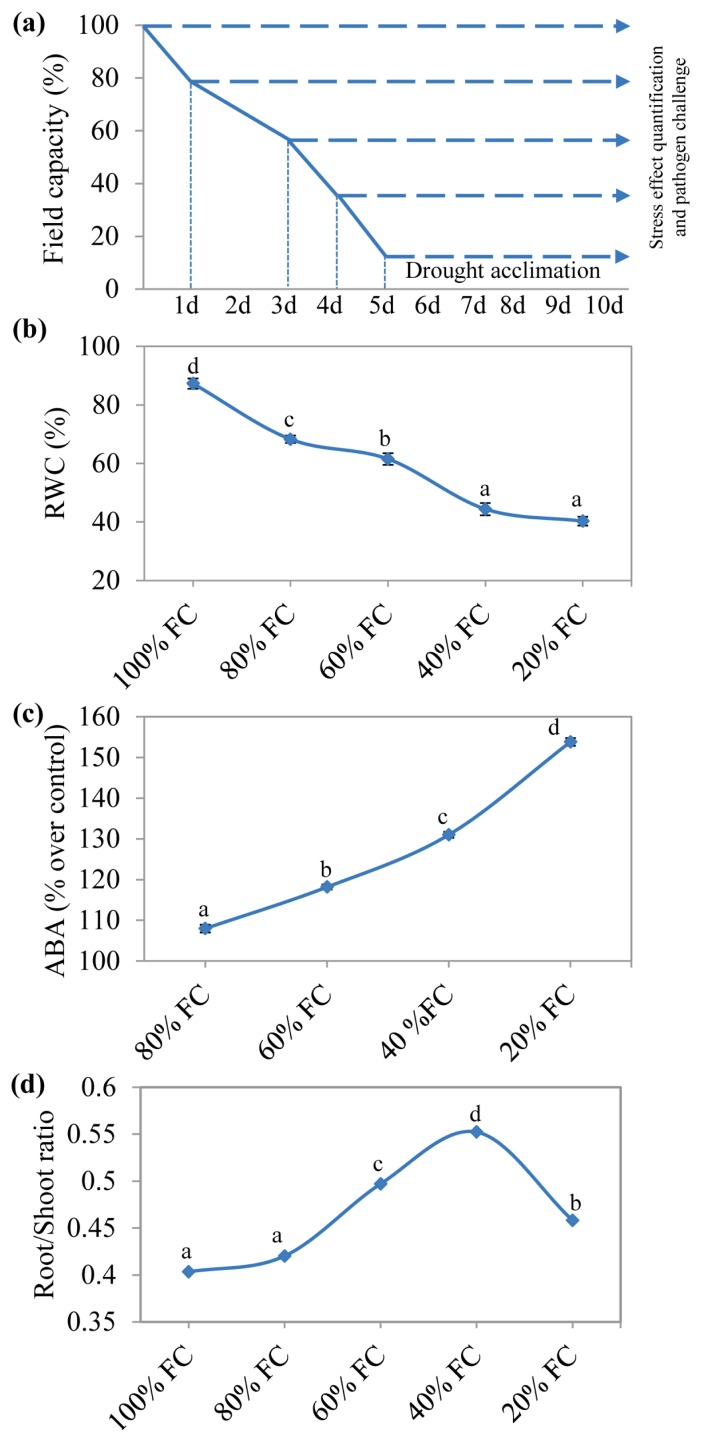
Drought acclimation of *N. benthamiana* plants. Drought stress was imposed on five-week-old plants following gravimetric approach. After all sets of plants reached specific FC’s, they were continued to be maintained at that level for five more days for acclimation (**a**). Stress levels were confirmed by stress-induced changes in relative water content (RWC) (**b**); ABA (**c**) and root-shoot ratio (**d**) at the end of acclimation period. Each bars represents standard error (*n* = 6). The one-way analysis of variance (ANOVA) was performed (*p* = 0.05), and letters above the data points indicate the significance and data points with the same letters are not significantly different.

To confirm precise drought stress imposition and acclimation of plants, relative water content (RWC), ABA and root-shoot ratio were measured at the end of stress period. Plants maintained at 100% field capacity (FC) showed nearly 90% RWC, whereas plants at 40% FC reached RWC of 45% (Figure 1a) confirming the cellular water deficiency in the stressed plants. Quantification of ABA in drought stress acclimated *N. benthamiana* plants showed increased ABA levels (Figure 1b). ABA content is known to increase under drought stress [42]. Reduction in cellular water levels decreases osmotic potential and RWC resulting in closure of stomata to limit water loss through transpiration. Stomatal closure as a result of reduced cellular water levels during drought is mediated by ABA [43]. Consistent with this, *N. benthamiana* plants undergoing drought acclimation showed reduction in RWC accompanied with increased ABA content as drought level increased. There was also considerable increase in root-shoot ratio in *N. benthamiana* plants maintained at different FC’s with maximum increase in root growth in plants maintained at 40% FC (Figure 1c). When subjected to drought stress, plants show differential response to root and shoot growth with rapid inhibition of shoot growth and continued root elongation. Root elongation under low water potential is considered to be an adaptation of plants to dry conditions as continued root elongation facilitates water uptake from the soil [44,45]. These physiological and biochemical changes confirms acclimation of *N. benthamiana* plants subjected to different drought stress levels.

### 2.2. Drought Acclimation Reduced *Sclerotinia sclerotiorum*-Induced Cell Death in N. benthamiana

**Figure 2:**
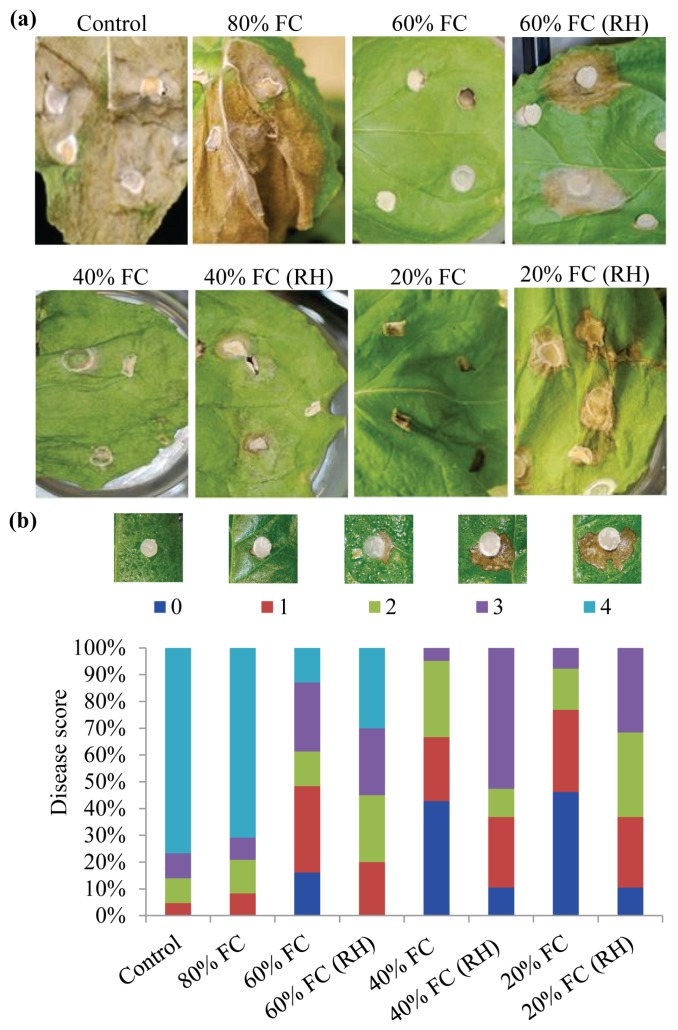
*Sclerotinia sclerotiorum-*induced cell death on *N. benthamiana* plants. Leaves of control and drought acclimated plants were inoculated with potato dextrose agar (PDA) plugs with actively growing *S. sclerotiorum* cultures. Photographs were taken at 4 dpi (**a**) and necrotic area was visually scored 0 (no cell death) to 4 (severe cell death) and expressed as percent of each score (**b**). Five independent plants were analyzed for each treatment (each with 30 inoculation spots). Experiments were repeated twice with reproducible results.

*S. sclerotiorum* is a broad host range fungus which can cause disease on *N. benthamiana* [46]. To determine the effect of drought acclimation on *S. sclerotiorum-*induced cell death, we inoculated drought acclimated *N. benthamiana* plants, at different FC’s for five days, with actively growing *S. sclerotiorum*. Drought acclimated *N. benthamiana* plants showed significantly reduced cell death symptoms caused by the fungus. In contrast, severe cell death was observed in non-acclimated control and plants maintained at 80% FC (Figure 2a,b). It is well known that relative humidity (RH) is an important factor that affects fungal infection and high RH facilitates better infection. Hence a set of plants were also infected with *S. sclerotiorum* and grown under high RH (80%) to determine whether the observed reduction in cell death was due to the intrinsic capacity of the drought acclimated plants or just mere reduction in RH on the leaf surface. Interestingly, there was no change in cell death or disease symptom development in plants maintained at 40% FC even under high RH when compared to plant maintained at 40% FC with low RH (Figure 2). These results suggest that acclimation to moderate drought stress (40% FC) has enhanced the capacity for effective activation of cellular defense response resulting in improved tolerance to subsequent infection of *S. sclerotiorum* and the observed tolerance is not due to lack of moist condition. As observed in plant-pathogen interactions, plants are also known to display priming-like reactions to abiotic stresses. It has been reported that biotic and abiotic stresses may interact both positively and negatively either by enhancing the resistance or increasing susceptibility to other stresses [4]. Recent evidences suggest that drought acclimation may enhance resistance to subsequent pathogen infection. In tomato, drought acclimation by three cycles of wilting and recovery enhanced the resistance to the fungus *Botrytis cinerea* [47]. In *Pinus yunnanensis*, plants which were exposed to mild water stress during winter showed enhanced resistance to a pathogenic fungus, *Leptographium yunnanense* [48]. A study in rose showed plant extract from drought stressed leaves controlled the black spot disease caused by *Diplocarpon rosae* through enhanced expression of pathogenesis-related proteins, peroxidase and accumulation of defense response elicitors [49]. Consistent with these reports, our results also suggest that drought acclimation of *N. benthamiana* plants might have primed a defense response resulting in enhanced tolerance to subsequent *S. sclerotiorum* infection.

### 2.3. Acclimation to Moderate Drought Stress Enhanced Tolerance to *Pseudomonas syringae* pv. *tabaci* in *N. benthamiana*

**Figure 3:**
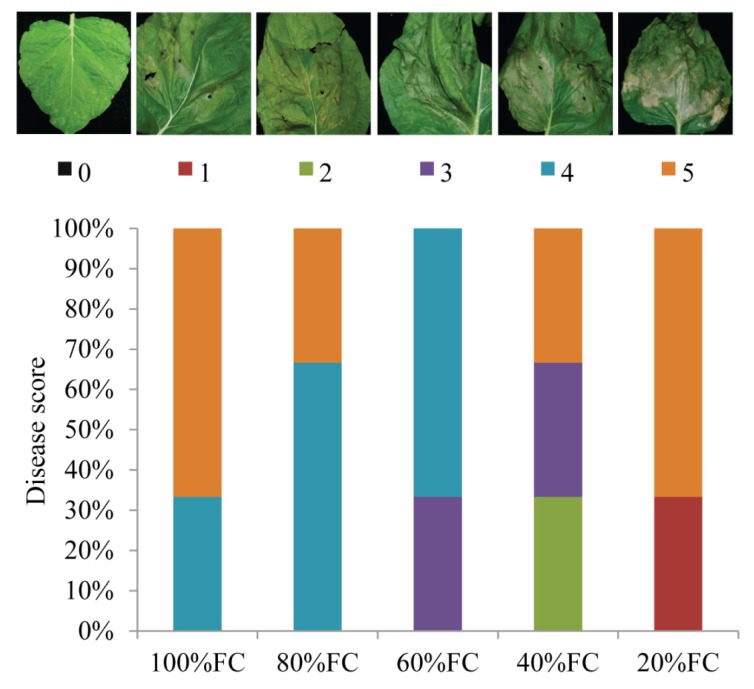
Disease-induced cell death in *N. benthamina* plants inoculated with *Pseudomonas syringae* pv. *tabaci*. Leaves of control and drought acclimated plants were inoculated with *P. syringae* pv. *tabaci* and pathogen-induced cell death was visually scored 0 (no cell death) to 5 (100% cell death at the inoculated spot) at 5 dpi. Three independent plants were analyzed for each treatment (each plant with 5 inoculation spots). Experiments were repeated twice with reproducible results.

**Figure 4:**
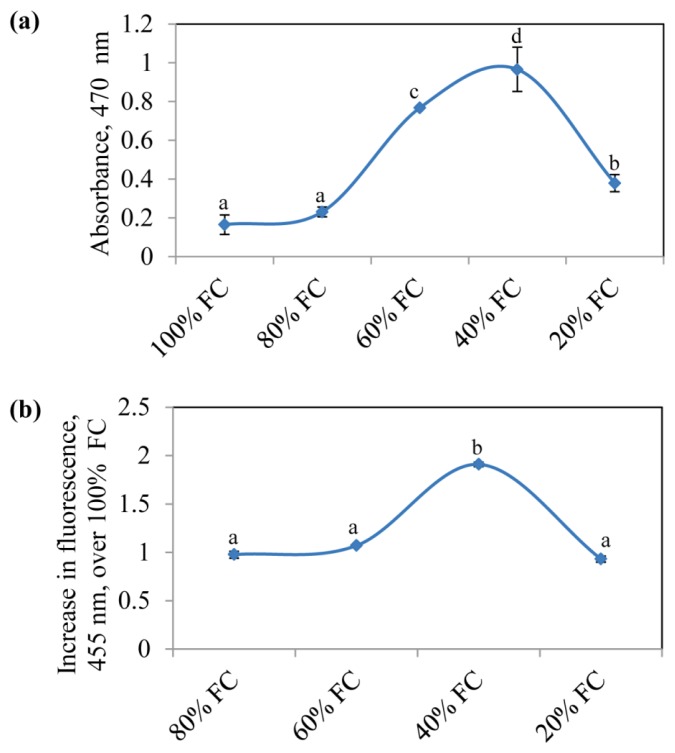

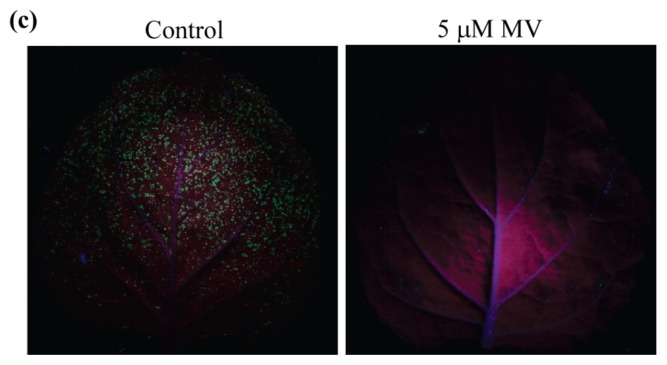
ROS levels in drought acclimated *N. benthamiana* plants. The ROS contents were measured in plants maintained at different FC’s at the end of stress period. The O_2_^−^ (**a**) and H_2_O_2_ (**b**) were quantified by XTT and scopoletin assay, respectively. Each bars represent the standard error (*n* = 6). The one-way analysis of variance (ANOVA) was performed (*p* = 0.05), and letters above the data points indicate the significance and data points with the same letters are not significantly different. (**c**) Visualization of bacterial growth in methyl viologen (MV) treated *N. benthamiana* leaves infected with *Pseudomonas syringae* pv. *tabaci*. Plants were sprayed with ROS inducer, MV, followed by infection with *GFPuv* expressing *P. syringae* pv. *tabaci*. Photographs were taken under UV light at 4 dpi.

To examine whether drought acclimation could also improve tolerance to plant-pathogenic bacteria, drought acclimated *N. benthamiana* plants were syringe (needless) infiltrated with a host pathogen, *P. syringae* pv. *tabaci* [50], which causes wildfire disease in tobacco [51]. Disease-induced cell death symptoms were visually scored at 5 days post-inoculation (dpi). More than 65% of the inoculated spots showed complete cell death and nearly 35% of the inoculated spots showed moderate cell death in plants maintained at 100% FC (Figure 3). The trend was very similar in plants maintained at 80% FC with more inoculated spots showing moderate cell death. Strikingly, the inoculated spots of plants maintained in 60% FC showed moderate to mild cell death and none of them showed complete cell death. Interestingly, nearly 30% and 70% of the inoculated spots showed complete cell death in plants maintained at 40% FC and severe drought stress of 20% FC, respectively (Figure 3). Surprisingly, when we quantified the bacterial growth in the inoculated spots of plants maintained in different FC’s, we did not see a significant difference at 3 dpi (data not shown). Disease symptoms induced by bacterial pathogens are not always correlated with bacterial growth as shown earlier [52,53]. From our results we suggest that acclimation of plants to moderate drought stress (60% FC) could limit the *P. syringae* pv. *tabaci*-induced disease development in *N. benthamiana*. At severe drought stress level (20% FC), the disease symptoms-induced by *P. syringae* pv. *tabaci* were quite similar to control (100% FC) plants suggesting the importance of moderate drought acclimation in imparting tolerance to bacterial pathogens. Lack of ROS induction and higher levels of ABA could be the reason for increased susceptibility of plants maintained at 20% FC (Figures 1c and 4a,b). Recent reports suggest that ABA plays antagonistic role in biotic stress signaling [30,54,55]. ABA treatment has been shown to antagonize SA, JA and ethylene defense signaling [56,57]. Negative effect of ABA on ROS mediated defense response has also been reported [58]. An ABA deficient tomato mutant plant, with impaired aldehyde oxidase activity which is required in ABA biosynthesis, accumulated H_2_O_2_ rapidly following infection by *Botrytis cinerea*, leading to a stronger defense response and greater resistance to the pathogen compared with the wild-type [58]. The susceptibility of *N. benthamiana* plants to bacterial pathogen maintained at 20% FC which also accumulated higher levels of ABA could be attributed to antagonistic effect of ABA on SA-mediated defense signaling.

### 2.4. Tolerance to Pathogen is Correlated with ROS Levels in Drought Acclimated Plants

We quantified the levels of O_2_^−^ and H_2_O_2_ in plants subjected to drought acclimation at different FC’s. The highest levels of O_2_^−^ and H_2_O_2_ were found in plants maintained at 40% FC. There was 6-fold increase in O_2_^−^ (Figure 4a) and 2-fold increase in H_2_O_2_ fluorescence_455_ at 40% FC (Figure 4b). At other stress levels (60% and 20% FC), which also showed considerable tolerance against both the pathogens, the O_2_^−^ levels were higher than non-acclimated control plants. It has been previously proposed that prior exposure of plants to abiotic stress alters pathogen resistance related activities that might result in enhanced resistance to subsequent pathogen challenge [59]. Though the mechanism or components involved in altered activities are not clear, there are evidences suggesting common connection points between biotic and abiotic stresses. Particularly, ROS generation has been suggested to be a central process in mediating the biotic and abiotic stress responses [30,60]. It is well known that the concentration of ROS increases during drought stress conditions [61]. During pathogen infection, plants rapidly generate ROS resulting in oxidative burst that limits pathogen spread through HR or cell death [35]. Our data shows that *N. benthamiana* plants acclimated at 40% FC drought stress enhanced tolerance to *S. sclerotiorum* and *P. syringae* pv. *tabaci*. The increased tolerance was correlated with higher O_2_^−^ and H_2_O_2_ levels in plants acclimated at 40% FC suggesting that high levels of ROS, very similar to oxidative burst under pathogen infection, might have helped reduce the disease development. It is not clear why the ROS levels decrease at severe stress condition (20% FC). In accordance with the reduced ROS levels, we saw reduced tolerance of *N. benthamiana* to tested pathogens at 20% FC when compared to moderate stress conditions (40% and 60% FCs).

To verify whether ROS generated in drought acclimated plants can reduce the pathogen infection, we used a ROS inducing chemical, methyl viologen (MV). We monitored the growth of *GFPuv* expressing *P. syringae* pv. *tabaci* pathogen [62] in MV sprayed *N. benthamiana* leaves. In light exposed plants, MV accepts electrons from photosystem I and transfers them to molecular oxygen generating superoxide in chloroplast [63]. In control plants intense bright green fluorescent spots representing bacterial colonies were observed under long wavelength UV light, while no or a very few green spots were visible on the leaves treated with MV suggesting the role of ROS in inhibiting bacterial growth (Figure 4c). High ROS levels in the plants maintained at 40% FC and complete prevention of bacterial growth in MV sprayed plants suggest that pre-generated ROS in drought acclimated plants might have primed the defense response against necrotrophic fungus, *S. sclerotiorum* and also to the bacterial pathogen, *P. syringae* pv. *tabaci*.

### 2.5. Drought Acclimation in *N. benthamiana* Induces Plant Defense Genes

**Figure 5:**
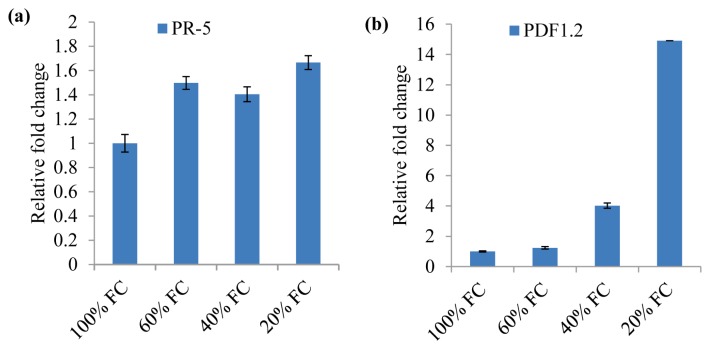
Increased expression of plant defense genes in drought acclimated plants. The relative mRNA expression levels of *PR*-5 (**a**) and *PDF1*.2 (**b**) were quantified by qRT-PCR in drought acclimated and control plants (100% FC). The fold change values were calculated using the 2^−ΔΔCT^ method and represented as changes in mRNA levels relative to 100% FC. *NbActin* was used as an internal control to normalize gene expression levels. The data are averages of two biologically independent experiments each consisting of three technical replicates.

Expression pattern of disease resistance marker genes was analyzed to understand the possible molecular mechanism for the enhanced defense capacity of drought acclimated plants. There was higher induction of *PR-5* (*pathogenesis-related protein-5*) and *PDF1*.2 (*plant defensin 1.2*) in drought acclimated plants (Figure 5). The *PR-5* gene encodes an osmotin and is induced through many signaling pathways including osmotic stress [64–66]. The *PDF1.2* gene encodes a plant defensin, and is induced by pathogen attack both locally and systemically via a JA/ethylene-mediated signaling pathway [67]. Studies have shown that several genes which are part of plant defense response to pathogen attack are also induced by osmotic stress but the role of these proteins in abiotic stress is not fully clear [68,69]. Our results suggest that pre-induction of pathogen defense responsive genes like *PR-5* and *PDF1.2* in drought acclimated plants might also contributed to the delayed disease development when challenged with *S. sclerotiorum* and *P. syringae* pv. *tabaci* in *N. benthamiana*.

## 3. Experimental Section

### 3.1. Plant Growth Condition

*N. benthamiana* seeds were germinated on flats filled with soilless potting mixture, Metro-Mix 830, BM7 (SUNGRO Horticulture Distribution, Inc., Bellevue, WA, USA). Three-week-old seedlings were transplanted to 10 cm diameter round pots (one seedling per pot) containing BM7. Plants were watered with fertilizer (20-10-20) containing soluble trace element mix (The Scotts Co., Marysville, OH, USA). The light intensity maintained in the greenhouse was 600 μE/m^2^/s with RH of 65%. The day/night cycles for temperature and photoperiod were 22 ± 2 °C/19 ± 2 °C and 14 h day/10 h night, respectively.

### 3.2. Drought Stress Imposition

Five-week-old plants were subjected to drought stress following gravimetric method of stress imposition. Soil moisture content at 100% field capacity (FC) was determined using the formula; Soil moisture content at 100% FC = [(SW − DW)/DW] × 100; Where SW-saturated soil weight taken after flooding the soil followed by overnight gravitational drainage; DW-oven dry soil weight. For stress treatments, soil water status was gradually brought down to specific FC (80%, 60%, 40% and 20%) by weighing the pots daily at a fixed time of the day [70]. Set of plants which reached specific FC early were maintained at that level by replenishing the lost water through evapotranspiration until the last set reaching 20% FC. Further, plants were maintained continuously at respective FC’s for five more days for acclimation (Figure 1a). Drought acclimated plants were then used for stress effect quantification and pathogen experiments. During the pathogen infection, plants were maintained at specified FC. A set of plants at 100% FC were used as non-acclimated controls.

### 3.3. RWC and Root-Shoot Ratio Measurement

RWC was determined following the protocol described by Flower and Ludlow, 1986 [71]. Briefly, after determining the fresh weight (FW), samples were immediately hydrated, by floating on de-ionized water in a closed Petri dish, to full turgidity for 4 h under normal room light and temperature and turgid weight (TW) was obtained. Samples are then oven dried at 50 °C until they reach constant weight to determine dry weight (DW). RWC was calculated using the formula:

RWC (%)=[(FW-DW)/(TW-DW)]×100

To estimate the root-shoot ratio, the aboveground and belowground parts were harvested separately and oven dried at 50 °C until they reach constant weight. The aboveground weight was considered as shoot and the below ground weight as root and the root-shoot ratio was calculated using following formula; Root-shoot ratio = Root dry weight/shoot dry weight.

### 3.4. ABA Quantification

Leaf tissue was ground to fine powder in liquid nitrogen and ABA was extracted in extraction buffer, containing 80% methanol with 100 mg/L butylated hydroxytoluene and 500 mg/L citric acid, in darkness for 16 h with constant shaking at 4 °C. The mixture was centrifuged at 5000 rpm for 5 min and the supernatant was diluted 10-fold with TBS buffer (50 mM Tris, 1 mM MgCl_2_, 150 mM NaCl, pH 7.8). The ABA concentration was quantified using the Phytodetek competitive ELISA kit (Agdia, Elkhardt, IN, USA) following the manufacturer’s instructions. A standard curve of different ABA dilutions [(+) *cis*/*trans* ABA; Sigma Aldrich, St. Louis, MO, USA)] was constructed to calculate the sample ABA concentrations.

### 3.5. *Pseudomonas syringae* pv. *tabaci* Inoculation and Scoring the Extent of Disease-Induced Cell Death

*P. syringae* pv. *tabaci* was grown in King’s B medium at 30 °C supplemented with rifampicin (25 μg/mL). Bacterial cells were harvested by centrifugation of overnight grown culture at 5000 rpm for 10 min, washed twice and re-suspended to the desired concentration (0.00002 OD) in sterile water. The bacterial suspension was used to inoculate leaves of drought acclimated *N. benthamiana* plants at respective FC’s using a needless syringe [50]. Plants were maintained continuously under drought for three more days and then re-watered. The disease-induced cell death symptom was scored at five days post-inoculation (dpi).

### 3.6. *Sclerotinia sclerotiorum* Inoculum Preparation and Plant Inoculation

*S. sclerotiorum* culture was initially grown on PDA (Difco, Sparks, MD, USA) medium and agar plugs (5 mm diameter) from actively growing regions were used as inoculum. Leaves of *N. benthamiana* plants maintained at different FC’s were inoculated with agar plugs and maintained at 65% RH. For increased RH, another set of plants inoculated with *S. sclerotiorum* were maintained at 80% RH chamber. All plants were kept at 22 ± 2 °C on a 14 h photoperiod at light intensity of 200 μE/m_2_/s. The disease symptoms were recorded at 5 dpi.

### 3.7. Quantification of ROS in Drought Acclimated Plants

Leaf tissue from drought acclimated plants, at a specified FC for 5 days, was used for quantifying O_2_^−^ and H_2_O_2_. Superoxide content was quantified by incubating leaf disks in 1 mL of K-phosphate buffer (20 mM, pH 6.0) containing 500 μM XTT (Polyscience Europe, Eppelheim, Germany) in darkness at 25 °C on a shaker. The increase in absorbance (A_470_) in the incubation medium was measured using spectrophotometer [72]. For determining H_2_O_2_ levels, leaf disks were pre-incubated for 30 min in 3 mL of K-phosphate buffer (20 mM, pH 6.0) to remove pre-formed H_2_O_2_ and were then incubated in 3 mL of the same buffer containing 5 μM scopoletin [7-hydroxy-6-methoxy-2*H*-1-benzopyran-2-one (Sigma Aldrich, St. Louis, MO, USA)] [73] and 3 μg/mL horseradish peroxidase in darkness at 25 °C on a shaker. The decrease in fluorescence (excitation: 346 nm, emission: 455 nm) in the incubation medium was measured using reagent blanks as reference.

### 3.8. Inoculation and Monitoring Bacterial Growth in Methyl Viologen Pre-Treated Plants

Initially a set of four-week-old *N. benthamiana* plants were sprayed with 5 μM MV. Six-hours later, plants were inoculated with *P. syringae* pv. *tabaci* expressing *GFPuv* [63] by vacuum infiltration. The inoculated plants were kept in greenhouse at 22 ± 2 °C. Two-days after inoculation, bacterial growth was visualized in leaves directly under UV light in a dark room. The photographs were taken using regular digital camera under UV light.

### 3.9. Real-Time Quantitative RT-PCR (qRT-PCR) Analysis

Total RNA was extracted from 100 mg of leaf tissue collected at the end of drought acclimation period using the RNeasy Plant Mini Kit (Qiagen, Valencia, CA, USA) following the manufacturer’s instructions. Two microgram of DNase treated total RNA was used for cDNA synthesis using SuperScript™ III First-Strand Synthesis System (Invitrogen™ life technologies, Grand Island, NY, USA). Equal dilution of cDNA was used as a template for qRT-PCR analysis using gene-specific primers (Supplementary Table S1) and the SYBR^®^ Green (Sigma Aldrich, St. Louis, MO, USA). The PCR reactions were run on Bio-Rad Thermal Cycler (Bio-Rad Life Science Research, Hercules, CA, USA) using the following program: 95 °C for 2 min, 95 °C for 15 s, 55 °C for 1 min for 40 cycles. The *NbActin* was used as internal control to normalize gene expression levels. Quantification of the relative changes in gene expression was performed using the 2^−ΔΔCT^ method [74].

## 4. Conclusion

The results from our study indicate that drought acclimation of *N. benthamiana* plants enhance tolerance to *S. sclerotiorum* and *P. syringae* pv. *tabaci*. The increase in ROS levels in drought acclimated plants could have primed the defense response in plants to subsequent pathogen infection very similar to second phase of oxidative burst in plant-pathogen interaction. Our study further strengthens the concept that ROS could be the central process connecting abiotic and biotic stress responses.

## Supplementary Information
