# Supplementary material for: Drought Stress Acclimation Imparts Tolerance to Sclerotinia sclerotiorum and Pseudomonas syringae in Nicotiana benthamiana
Source: Int J Mol Sci. 2013 May 2;14(5):9497–513. doi: 10.3390/ijms14059497 (PMC3676796; doi:10.3390/ijms14059497)
Supplement: Supplementary file 1 [file ijms-14-09497-s001.pdf]

Supplementary Information

Table S1. Primers used for quantitative RT-PCR analysis.

Gene	TIGR gene index	Forward primer (5'–3')	Reverse primer (5'–3')
NbPR-5	TC21926	GTGGGCGCCCTGGAAGAGT	CACGCGACAGTACATAAAAGTT
NbPDF1.2	TC23349	CTTCAAGCAAAGCTGCAGCCAAAG	CTATGCACTAAGCCATGTGTGTTTG
NbActin	GO608298	ACATGTAACCACGCTCGGTAAGGA	TGTGTTGGACTCTGGTGATGGTGT

© 2013 by the authors; licensee MDPI, Basel, Switzerland. This article is an open access article distributed under the terms and conditions of the Creative Commons Attribution license (http://creativecommons.org/licenses/by/3.0/).
